# Reducing Physical Health Inequalities: A Community Mental Health Clinic Quality Improvement Project

**DOI:** 10.1192/bjo.2025.10327

**Published:** 2025-06-20

**Authors:** Murtala Abdussalam, Tanisha Smith

**Affiliations:** 1 North Staffordshire Combined Healthcare NHS Trust, Stoke-on-Trent, United Kingdom; 2 Royal Stoke University Hospital, Stoke-on-Trent, United Kingdom

## Abstract

**Aims:** People with severe mental illness (SMI) experience greater physical health inequalities, poorer health outcomes and significantly lower life expectancy (15–20 years) than the general population. Despite National and local guidelines on routine physical monitoring to mitigate these inequalities, the practice in this clinic is inconsistent, often fraught with delays and inefficiencies. This quality improvement project (QIP) aims to achieve full compliance with monitoring guidelines to 100% over 8 weeks, enhancing patient safety and outcomes.

**Methods:** A baseline audit on compliance with physical health monitoring guidelines was conducted by reviewing the local and national guidelines, clinic records, the monitoring log, and Resident Doctors’ outstanding reviews list. A semi-structured staff interview and reviewing GP correspondence identified key barriers to compliance. Three changes were introduced using the PDSA framework (plan, do, study, act); first staff training on Kardia ECG interpretation; then weekly logging and mop-up of outstanding ECG (electrocardiogram) and blood test results; and third, streamlining workflow by improving cover and swap during annual leaves to ensure continuity.

**Results:** There was a significant improvement in compliance with physical health monitoring during the project. Outstanding blood test results decreased from 140 to 24, and pending ECG results from 16 to 1 in four weeks. By eight weeks, full compliance (100%) was achieved. The weekly mop-up proved the most effective intervention in clearing the backlog while staff reported that training in Kardia enhanced their confidence in conducting the reviews.

**Conclusion:** The QIP illustrates that improving physical health intervention requires targeted interventions that can result in improved physical health monitoring in community mental health centres. Sustaining this improvement requires enhancing staff skills and confidence, greater team collaboration and coordination of planned staff absences through appropriate swapping and cover arrangements. Future work will focus on developing a centre-specific guide, improved induction and shadowing for Resident Doctors and involving them in job planning.

